# Cheetah Reunion – The Challenge of Finding Your Friends Again

**DOI:** 10.1371/journal.pone.0166864

**Published:** 2016-12-07

**Authors:** Tatjana Y. Hubel, Justine Shotton, Simon D. Wilshin, Jane Horgan, Rebecca Klein, Rick McKenna, Alan M. Wilson

**Affiliations:** 1 Structure and Motion Laboratory, Royal Veterinary College, Hatfield, UK; 2 Cheetah Conservation Botswana, Kgale Siding Park, Plot 1069-KO, Gaborone, Botswana; Institute of Zoology, CHINA

## Abstract

Animals navigate their environment using a variety of senses and strategies. This multiplicity enables them to respond to different navigational requirements resulting from habitat, scale and purpose. One of the challenges social animals face is how to reunite after periods of separation. We explore a variety of possible mechanisms used to reunite the members of a cheetah coalition dispersed within a large area after prolonged separation. Using GPS data from three cheetahs reuniting after weeks of separation, we determined that 1) the likelihood of purely coincidental reunion is miniscule 2) the reunion occurred in an area not normally frequented 3) with very little time spent in the region in advance of the reunion. We therefore propose that timely encounter of scent markings where paths cross is the most likely mechanism used to aid the reunion.

## Introduction

Separating and reuniting is common behaviour for many social animals, with reasons ranging from foraging and mating to the gradual weaning of adolescents [1]. In many species, mothers leave their young alone for periods of time before reuniting with them at the location of separation [2–4]. Leaving young or group members behind requires the ability to locate one another again. Returning to known locations such as breeding grounds and dens requires excellent navigational skills [5–7].

Animals use a diversity of senses and strategies to master the challenges of navigating their environment. Navigation can be based on either egocentric or geocentric systems of reference or a combination of both [8]. Animals that predominantly use egocentric navigation rely on keeping track of their position by estimating the direction and distance travelled (path integration/dead reckoning) [9–11]. In contrast geocentric navigation relies on obtaining positional information based on cues such as celestial information [12–14], landmarks [15], magnetic fields [16] and smell [17]. When breeding in large colonies, finding one’s own offspring among others poses another great challenge and is aided by scent and identification calls [18]. Several species are known to use chemo sensing to search out females [4, 19, 20].

While finding one’s way to a particular location is challenging and a topic of intense research, even more challenging and less understood are the mechanisms of reuniting with group members or finding potential mates when both parties roam across large ranges. For example, members of a lion pride do not always behave as a cohesive unit, but frequently split and scatter widely [21]; equally, male cheetah in bonded coalitions split up occasionally (http://org.elon.edu/ncwildcat/nc-cheetahs/day_in_life.html). This behaviour was verified in the coalition used in this study using camera traps beginning in April 2015. Compared with lion, whose roars carry up to 4 km [21], cheetah with their relatively soft chirps that only carry a few hundred meters [21], cannot use their calls to regroup when widely separated [22] and it has been proposed they might have to rely on chance [22].

Another aspect of navigation is to avoid predators or rivals in order to increase the likelihood of survival. These avoidance behaviours have been documented for a wide range of species [23–25]; in these cases animals actively move away from risky locations when clues (visual, olfactory, tactile or auditory) indicate the presence of rivals and predators [24]. Game theoretic models have been used to examine spatial use by predator and prey species or multiple competing predators [23, 26–29]. Early work has suggested apex predators move towards prey resources such as grazing lands or water sources, with prey balancing predation risk against resource gathering [23, 29]. Recent, more complex, multi-predator models imply a greater variety of viable strategies for prey animals [27]. However, while these avoidance scenarios have been investigated to some extent, relatively little is known about large scale reunion methods.

To shed light on possible methods used to find group members in a large scale environment, we used fine-scale global positioning (GPS) data to analyse the separation and reunion process between members of a cheetah coalition in the vast Ghanzi farmland region of Botswana.

## Material and Methods

The coalition of three cheetahs (most likely brothers) used for this analysis were among the subjects of a larger study. Camera trap evidence indicated that a fourth cheetah, who was not collared, was also part of the study coalition.

Collars fitted with a programmed drop-off unit were deployed from July 2014-January 2015 (approved by RVC Ethics & Welfare Committee and under Botswana Department of Wildlife and National Parks research permit EWT8/36/4). Cheetahs were cage-trapped at known marking trees and fitted with collars. The collars switched dynamically between different operational modes depending on the movement detected by an associated inertial measurement unit (IMU) that records the body’s angular velocities and linear accelerations [30]. GPS fixes were recorded hourly for stationary animals, every five minutes for moving animals and at 5Hz for running animals. All calculations were conducted using Matlab (Mathworks Inc., Natick, MA). For the purpose of this analysis all data were interpolated to 5-minute fixes at fixed time points (S1 Table). The accuracy of GPS fixes was within approximately 5 m [30]. The distance between individuals was calculated for all time points. The home range was calculated using the basic alpha shape with a probe radius of 5000m. During the 6.5 month tracking period, the coalition of three cheetahs remained together, with the exception of one period of 31 consecutive days for which they separated. During the period of separation, two cheetahs travelled together and were treated as one single unit represented here by one of them as they stayed so close together. The third member of the coalition was treated as a separate unit.

The probability of meeting by chance in the home range was calculated by assuming both groups have to occupy the same space. Assuming that on a given day the probability of being within a certain radius of a point is the ratio of the area of that circle to the total area, the probability of being within the circle at any given day is:

$$q=A_{1}/A_{2}$$ (1)

where *q* is the probability, *A*_*1*_ the area of the circle and *A*_*2*_ the total area.

Taking GPS accuracy into account, a 5 m radius was chosen for *A*_*1*_ (*A*_*1*_ = 78.5 m^2^) and A_2_ was the home range area (819200000m^2^).

The probability of never being inside *A*_*1*_ over a certain number of days (*d*) is:

$${\left(1-q\right)}^{d}$$ (2)

The probability that one is ever inside A_1_ is one minus the probability of never being inside that area. So the probability of ever being inside the area is:

$$1-{\left(1-q\right)}^{d}$$ (3)

It was presumed that cheetah can detect each other at a certain, but undefined, proximity. Although cheetah have an excellent sense of smell and very good hearing they rely primarily on vision during hunting [31, 32]. Cats are known for their night vision but are rather nearsighted [33]. However, unlike other felids, cheetah are crepuscular and have a higher concentration of nerve cells in the centre of their eyes, called a "visual streak", which significantly enhances the sharpness of their vision, so they can spot moving prey at a distance up to 2 km [34]. However, the detection distance will depend on factors such as vegetation and terrain. Ghanzi farmland is primarily made up of low tree and shrub savannah, which will obstruct the view. Using an admittedly somewhat arbitrary detection radius of 300 m the probability of meeting was recalculated.

Not all areas of the home range were used equally; therefore the 31 days were permuted randomly (over 10,000 combinations) in order to calculate a more conservative chance of meeting.

## Results and Discussion

**Fig 1:**
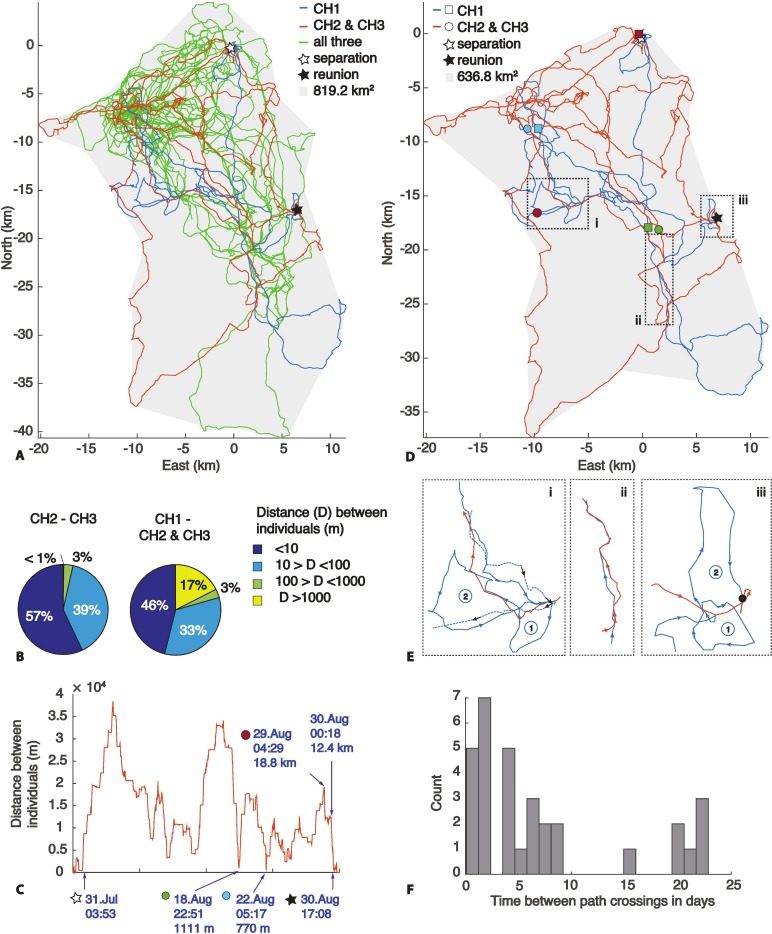
A) GPS traces of the three cheetahs over the course of 6.5 months. Path colored based on how may animals travelled together at the time (green: all three, red: group of two (CH2 and CH3), blue: one individual (CH1), grey shading: home range). B) Percentage of time the individuals spent within X meters of each other over 6.5 months. C) Distance between the lone individual (CH1) and the other two (CH2 & CH3) at any given time during separation (markers correspond with locations in D). D) GPS traces during time of separation, with locations of separation, minimum distances, maximum distance shortly before reunion and reunion displayed (see markers in C). E) Zoom windows (i, ii, iii) into (D) when the two groups followed the path of the other temporarily within 4.5 days. Potential search loops are identified by (1) and (2) with a successful reunion on the 30^th^ of August (iii), when CH1 crossed the same path within 2.4 hours of the other two and reunion occurred 9.5 hours later. F) Histogram of time past between the two groups encountering each other’s tracks.

The positions of the three cheetahs during the 6.5 months of GPS recording is displayed in Fig 1A. To the best of our knowledge this is the first study to obtain high-resolution data for multiple individuals in a cheetah coalition, which enables tracking of their detailed movements relative to each other.

Most of the time, the individuals stayed within 100 m of each other (Fig 1B); two of the members (CH2 and CH3) were separated by more than 1000 m for less than 1% of the time. The third member (CH1) split from the other two for 31 consecutive days (Fig 1C and 1D, S1 Movie) and then reunited with them. He stayed behind at a marking tree location next to an artificial waterhole, and remained within 1.4 km^2^ of that position for the first 8 days, before moving on. The reason for this separation is unknown and no hunts were recorded by CH1’s collar during this time. Camera traps confirm that CH1 was alone at the waterhole after the departure of the others; the fourth (uncollared) cheetah was captured on a camera trap together with the other two on the day before reunion. From the collar and camera trap data, we infer that the fourth cheetah travelled with CH2 and CH3, and CH1 most likely reunited with all three cheetahs on the 30th of August.

Home range for the three collared cheetahs was estimated to be 819 km^2^ (Fig 1A), which is a medium sized home range compared to previous studies in similar farming environments, where they varied from an average of 1600 km^2^ in Namibia [35], to 668 km^2^ in Botswana [36], to only 190–310 km^2^ in South Africa [37]. The three cheetahs used most of their home range (637 km^2^) during separation (Fig 1D) with no apparent demarcation. The likelihood of randomly occupying the same space (ie being within 78.5 m^2^; based on 5 m GPS accuracy [30]) within such a large area is infinitesimally small, 9.58 x10^-6^% and rising to 2.97 x10^-4^% over the course of 31 days. Assuming detection proximity of 300 m within which they are able to identify each other increases the chance to 1.06%. Permutating the days randomly estimates a 3.5% chance of the two groups being within 300 m of each other over the entire 31-day time period. In reality the two groups came within 770 m and 1110 m of each other on two occasions without resulting in a reunion (Fig 1C). Furthermore, the reunion did not occur in the most frequented area (Fig 1A) and neither of the groups spent more than 24 hours in the area before reunion. Also, the area where the reunion occurred was visited only once by one of the groups 25 days before the reunion, discounting the notion that marking tree visits by the two groups in rapid succession leads to reunion. No further visit to that location occurred after the reunion during the remaining 4 months of the study period.

Among the felids, lion are the only true social cats. They live in prides and separate and reunite frequently [21]. Long distance calling is thought to play a major role in their ability to find each other again [21]. However, calling out could have detrimental effects if one needs to stay hidden from rivals or other predators. Single female lion and nomadic males avoid roaring in circumstances when it is beneficial to conceal their presence [38, 39]. Attracting unwanted attention can potentially be deadly. Larger predators often kill smaller competitor species and playbacks of African wild dog vocalisation have shown that lion actively move towards their location [40], while African wild dogs move away from lion calls [41]. Considering the vulnerability of cheetah to a range of predators it is unlikely that long periods of vocalisation are used for long distance communication and the chirping sound carries far less than lion roars [21] or wolf howls [42].

Felids have an excellent sense of smell and there are anecdotal observations of lion following fresh trails of other lion for several kilometers using their nose [21]. Like other cats, cheetah are able to identify individuals by their unique scent [4] and spend considerable time and effort scent marking on landmarks. Scent is a major communication tool, reducing aggressive encounters between groups [4] and aiding the location of females in oestrous [4]. It is therefore reasonable to assume that it is also a useful tool for locating missing group members. The scent of cheetah is missing organosulfur components present in other cats’ scent, possibly as a mechanism to avoid detection by larger predators [43].

During their separation, the tracks of the two groups intersected on 31 occasions (multiple encounters within 4 hours were counted as the same event) (Fig 1F). They encountered each other five times within 24 hours and twice within 10 hours (2.4h, 7.2h). Reunion occurred after CH1 crossed the trail within 2.4 hours of the other group, which remained stationary in the area. However, unlike the observations of lion that directly tracked pride members by following a scent trail, CH1 executed what appeared to be a search pattern of two loops before the reunion, possibly initiated by picking up the scent of the other group (S2 Movie). Other instances when the paths of the two groups intersected were identified and are magnified in Fig 1E. A similar loop pattern was evident once before; however, the scent mark would have been five days old by the time of the encounter and the incentive for inducing such a search pattern after this time is unknown. The time scent lasts is speculative (24 hours to 2 months have been proposed for different species [4]). The sensitivity of the cheetah’s olfactory sense compared with lion or canids is not known, and while clearly they can detect scent marks, so far there has been no evidence that they can follow a track purely by smell. However, once a fresh scent mark is detected in an area, loop pattern searches and vocalisation could be useful tools to enhance the likelihood of reunion.

Although this case study examines only a single reunion event, we believe the data allow a unique insight into the challenges and possible mechanisms of the reunion of two moving parties in large areas. Obtaining a dataset with such high spatial resolution on wild African predators is extremely challenging given the welfare, expense and logistical considerations, as well as the unpredictability of separation and reunion (once in 6 months here).

This study has shed new light on the detailed movement and ranging behaviour of members of a cheetah coalition in farmland in Botswana. The high resolution data has enabled us to explore the mechanisms of reunion by excluding mechanisms that have definitely not been used. Analysis of the data revealed a number of interesting findings: 1. most of the home range was used during separation, 2. there was only a very small chance that reunion occurred by chance, 3. reunion occurred in a region of low use, 4. it occurred after both groups had spent only hours in the region, 5. and within hours of encountering the route travelled by the other party, 6. reunion followed a possible search pattern. We are therefore led to suggest that the reunion most probably resulted from coincidental appearance in the same region of the home range, aided by timely encounter of scent marks and possibly by vocalisation when in close range [44].

## Supporting Information

S1 Movie GPS tracks of all cheetahs during time of separation. Cheetahs split into two groups with CH1 (Aragon) as one unit and CH2 and CH3 (Gimli and Legolas) as the second unit. The center of the home range was chosen as point of origin. Path overlap when cheetahs travelled together.
(MOV)

S2 Movie GPS tracks of all cheetahs shortly before reunion. (MOV)

S1 Table Dates, UTC times and position data (latitude, longitude) for the three cheetah. Data interpolated to 5-minute fixes at fixed time points.
(TXT)
